# Canine stress analysis of two clear aligner attachment designs: A finite element study

**DOI:** 10.6026/973206300221593

**Published:** 2026-03-31

**Authors:** Y Sarita, Sunil Sharma, Anurag Tiwari, Jayant Chandrakar, Nishu Singh, Amit Kumar

**Affiliations:** 1 Department of Orthodontics and Dentofacial Orthopedics, NIMS Dental College & Hospital, NIMS University Rajasthan, Jaipur, India; 2 Department of Oral & Maxillofacial Surgery, NIMS Dental College & Hospital, NIMS University Rajasthan, Jaipur, India; 3 Department of Orthodontics and Dentofacial Orthopedics, Maitri College of Dentisrty and Reserch Centre, Anjora (Durg) Chhattishgarh, India; 4 Department of Conservative Dentistry and Endodontics, Hazaribag College of Dental Sciences and Hospital, Jharkhand, India; 5 Department of Oral & Maxillofacial Surgery, Sarjug Dental College and Hospital, Darbhanga, Bihar, India

**Keywords:** Clear aligners, composite attachments, finite element analysis (FEA), orthodontic tooth movement, biomechanics

## Abstract

A finite element analysis compared the stress distribution profiles of a single rectangular attachment against a dual semicircular configuration to resolve the biomechanical intricacies of achieving bodily canine translation with clear aligners. Hence, three-dimensional model of the maxilla was generated to simulate stress propagation across the teeth, periodontal ligament and alveolar bone under standardized loading conditions. Von mises stress assessment revealed that the rectangular geometry induced markedly higher stress concentrations (0.1922 MPa), suggesting an elevated risk of root resorption and appliance fatigue. Conversely, the dual semicircular design exhibited enhanced biomechanical efficiency, demonstrating a 27% reduction in stress magnitude (0.1411 MPa) and minimized localized pressure. These data advance clinical understanding by establishing dual semicircular attachments as a safer, more effective modality for predictable canine movement.

## Background:

In recent years, there has been a significant rise in adult patients seeking orthodontic treatment [1], with aesthetics playing a crucial role in their choice of appliances. This demand has driven the development of more discreet and comfortable alternatives to traditional braces, such as clear aligners. These transparent, removable trays offer an appealing solution due to their nearly invisible appearance, smooth texture and convenience. Unlike fixed appliances, clear aligners use a series of custom-made trays to apply controlled forces, gradually guiding teeth into proper alignment. While they are particularly effective for mild to moderate cases, their ability to achieve complex tooth movements remains a challenge. Nevertheless, ongoing advancements in materials and digital treatment planning continue to expand their potential, making clear aligners an increasingly popular [2] option in modern orthodontics. Composite attachments in clear aligner therapy are small, precisely designed composite structures bonded to teeth to enhance aligner retention and force transmission from clear aligners to teeth [3]. Their geometry, size and positioning are optimized to generate specific force couples and moments for controlled tooth movement-addressing limitations of aligner materials, which exhibit stress relaxation [4] and force decay over time. By strategically spacing attachments, clinicians can influence the magnitude of forces and moments while minimizing excessive pressure that could lead to aligner deformation. However, the viscoelastic nature of current polymers still poses challenges in maintaining consistent force delivery, necessitating careful planning to balance effective tooth movement with material limitations. FE models applied to new aligner-based orthodontic techniques allow a better understanding of their theoretical performance, allowing us to this understanding with regard to the clinical setting. For clear understanding of thermoformed aligner orthodontics, two fundamental differences between bracket- based and aligner-based biomechanics must be recognized. For several decades, 3D simulation analysis was widely used in the field of dental research by building a hypothetical 3D finite element model assuming the dental treatments and surgical conditions. Specifically, the finite element analysis (FEA) is non-invasive and is a virtual model, it has the advantage of being able to predict the results without direct application in patients. In addition, it allows analysis to be conducted by simulating the procedural method and environment that are difficult to apply in actual clinical setting [5]. Using a mathematical model derived from the CAD in three dimensions, finite element (FE) models contribute to the understanding of biomechanics of the orthodontic devices as they permit the estimation of the stresses generated within the different tissue structures, such as alveolar bone, periodontal ligament (PDL) and teeth, during the treatment. Therefore, it is of interest to show the loading and displacement patterns determined models.

## Materials and Methods:

The designing of 2 FEM models were done in solid works software and simulation in ANSYS software version 14 to evaluate the stress distribution in maxillary right canine with two different sizes of attachment. Finite element model of all maxillary teeth including the extracted first pre-molar, periodontal ligament, alveolar bone, clear aligner, two types of attachments- rectangular and semicircular, were constructed using mechanical elastic properties of the materials such as young's modulus of elasticity and Poisson's ratio as shown Table 1 (see PDF). All the materials were homogenous, isotropic and linearly elastic. To determine the stress magnitude, a 3-dimensional model of maxillary canine with attachments, was constructed along with periodontal ligament and alveolar bone and the interface between the alveolar bone socket and root surface was 0.2 mm as the width of periodontal ligament (PDL) is 0.2 to0.35 mm. The final meshwork consisted of total 433369 nodes (aligners-1498, rectangular attachment-688 & semicircular attachment- 617) and 1657957 elements (aligners-4818, rectangular attachment-2676 & semicircular attachment-2393). The attachments were placed at the center of the labial surface of maxillary right canine along with clear aligner with composite resin adhesive layer which had mean thickness of 0.2mm.

Based on size of attachment FEM models were divided into two groups:

[1] Group I: The FEM models of maxillary dental arch in which the large one rectangular attachment is placed on the labial surface of canine.

[2] Group II: The FEM models of maxillary dental arch in which the two small semilunar attachments are placed on the labial surface of canine.

## Results and Discussion:

Our study evaluates the stress distribution of two composite attachment designs-rectangular and semicircular-in facilitating controlled bodily movement of upper canines during clear aligner therapy. The findings provide critical insights into force transmission, displacement efficiency and stress distribution, offering clinically relevant guidance for attachment selection. Stress distribution and biomechanical efficiency. Von mises stress analysis revealed that rectangular attachments concentrated 36% higher stress (0.1922 MPa) than their semicircular counterparts (0.1411 MPa) Table 2 (see PDF), Figure 1 (see PDF), with similar trends observed in tension and compression stresses. This elevated stress magnitude, while facilitating greater displacement, may increase risks of root resorption, aligner fatigue or patient discomfort. Semicircular attachments, by contrast, demonstrated 26-27% lower stress levels, suggesting a more biomechanically efficient design that minimizes localized pressure while maintaining therapeutic efficacy. Clinical implications for attachment selection; the data underscore a fundamental trade-off: rectangular attachments maximize displacement at the expense of controlled movement, making them suitable for initial alignment phases requiring significant tooth repositioning. Conversely, semicircular attachments optimize controlled bodily movement; rendering them ideal for finishing stages where precision and root stability are paramount. Their rounded geometry also offers aesthetic and comfort advantages, enhancing patient compliance in visible dental zones. This FEM study found that the mesial occlusal-mesial cervical attachment configuration optimizes mandibular canine movement in clear aligner treatment, increasing strain by 33.1% for distal tipping and enhancing bodily movement. Results align with Melsen (2011) on attachment efficacy and frost's bone remodeling theory. The study highlights the need for precise attachment design but acknowledges modeling limitations [6]. The FEM study found clear aligners with attachments rotate lower premolars best at 1.2°C activation. Single attachments worked most efficiently while 3°C activation caused excessive stress. Attachments proved essential for control, showing aligners alone were ineffective. The research recommends limiting rotation to 1.2°C per aligner for safe, predictable movement [7, 8]. The FEM evaluates the forces and moments produced by clear aligners, showing they are comparable to fixed braces for movements like incisor torque, premolar derotation and molar distalization. However, the flexibility of the plastic and its fit from incisal to gingival margins can limit precision, especially for maxillary laterals and cuspids. This highlights biomechanical challenges in achieving predictable tooth movement with aligners [7]. The FEM analysis evaluated the biomechanical effects of attachments on clear aligners, focusing on incisor extrusion. It found that adding a palatal-side attachment improved extrusion efficiency by enhancing force application. Another FEM study analyzed canine distalization with aligners and composite attachments, showing that attachments help generate controlled forces for predictable tooth movement. These studies highlight that attachments optimize aligner biomechanics by improving force transmission and movement accuracy, particularly for challenging movements like extrusion and rotation. However, clinical validation is needed to confirm these findings [9, 10]. The FEM analysis evaluated different auxiliary-aligner designs for maxillary central incisor extrusion. It found that attachments on the palatal side improved extrusion efficiency by enhancing force application. The study highlights that attachment design significantly impacts aligner performance, with horizontal attachments proving more effective than optimized ones for extrusion. These findings align with clinical trial results showing better predictability with horizontal designs [11]. Another article determining optimal orthodontic forces for maxillary canine movement using finite element analysis (FEA), with emphasis on stress and strain in the periodontal ligament (PDL) during various tooth movements like translation, tipping, extrusion and rotation. The findings highlighted the importance of balancing PDL stress (0.47-12.8 KPa) and strain (>0.24%) to avoid root resorption while ensuring efficient tooth movement [12]. The findings highlighted aligners' limitations in controlling complex movements (e.g., extrusion accuracy: 30%) and emphasized the need for auxiliaries (attachments, IPR) to improve predictability [7, 13]. Recent investigations further confirm these constraints, emphasizing the critical gap between programmed and achieved root movement [14]. To address these deficiencies, further FEM studies evaluated the impact of attachment geometry-specifically semicircular, vertical rectangular and horizontal rectangular designs-on the bodily distalization of maxillary canines. While the specific shape of the attachment showed minimal variation in efficacy, the presence of any attachment was found to be essential for preventing the tipping and rotation observed in aligners without auxiliaries [15, 16]. Additionally, the initial force systems acting on the dentition were found to be heavily influenced by pre-treatment canine angulation. Due to the relatively low rigidity of the aligner material (0.45 mm thickness; 2 GPa Young's modulus), torque loss and mesial slipping of anterior crowns were observed. It was established that distally tipped canines require specific anti-tip mechanics to ensure bodily movement, whereas lateral incisors and premolars bear the highest force loads, necessitating rigorous retention protocols [17].

## Conclusion: 

The analysis confirms that attachment configuration is a critical determinant of treatment biomechanics, where rectangular designs offer superior force magnitude but higher stress risks, while semicircular variants present a balanced approach that facilitates predictable bodily movement with mitigated biomechanical concerns. Finite element analysis further demonstrated that increasing the magnitude of simultaneous canine retraction during maxillary anterior en-masse retraction significantly enhances incisor root control by counteracting uncontrolled lingual tipping, although this biomechanical advantage is accompanied by a concomitant increase in posterior anchorage loss. Consequently, optimizing therapeutic outcomes requires a multifaceted approach that integrates phase-specific attachment selection with precise staging of differential canine and incisor movements to achieve effective anterior retraction while preserving posterior anchorage integrity.
